# Utilization of Antireflux Mucosectomy and Resection and Plication: A Novel Approach for the Management of Recurrent Gastroesophageal Reflux Disease after Prior Nissen Fundoplication or Transoral Incisionless Fundoplication

**DOI:** 10.3390/jcm11237141

**Published:** 2022-12-01

**Authors:** Navkiran Randhawa, Ahamed Khalyfa, Mahnoor Khan, Christopher Roebuck, Mahnoor Inam, Kamran Ayub

**Affiliations:** 1 Franciscan Health Olympia Fields, 20201 South, Crawford Ave, Olympia Fields, Chicago, IL 60461, USA; 2 Icahn School of Medicine at Mount Sinai, 1 Gustave L. Levy Pl, New York, NY 10029, USA; 3 RxEconomics LLC, 1350 Mccormick Rd Ste 705, Hunt Valley, ML 21031, USA; 4 Southwest Gastroenterology, 9921 SW Hwy, Oak Lawn, IL 60453, USA; 5 Silver Cross Hospital, 1900 Silver Cross Blvd, New Lenox, IL 60451, USA

**Keywords:** antireflux mucosectomy, resection and plication, gastroesophageal reflux disease, Nissen Fundoplication, Transoral Incisionless Fundoplication

## Abstract

**Background**: Nissen Fundoplication (NF) and Transoral Incisionless Fundoplication (TIF) are established procedures for the treatment of gastroesophageal reflux disease (GERD). However, the surgically induced plication can loosen over time. This multicenter study aims to evaluate the safety and efficacy of Antireflux Mucosectomy (ARMS) and Resection and Plication (RAP) in symptomatic patients with prior NF or TIF that has become loose. **Patients and methods**: Eighteen patients were enrolled in the study. Ten had prior TIF, while eight had prior NF. Half of these patients had a Hill Grade 3 Valve while the other half had a Hill Grade 2 valve. Endoscopic submucosal dissection (ESD) was performed in six patients, while endoscopic mucosal resection (EMR) was performed in twelve patients. A follow-up endoscopy was performed at 4–12 weeks. **Results**: At follow-up, 11 patients had a Hill Grade 1 valve, and seven patients had a Hill Grade 2 valve. All patients had improvement in symptoms for up to 32 months. **Conclusions**: In this pilot study, ARMS/RAP appears to be an effective option in patients who had prior NF or TIF with recurrent GERD symptoms.

## 1. Introduction

Gastroesophageal reflux disease (GERD) is a prevalent digestive disorder affecting nearly 20% of the United States’ population [1,2]. Acid regurgitation and heartburn are common manifestations of GERD; however, diagnosis is usually dependent on a combination of clinical symptoms, objective endoscopy assessment, and pH monitoring. Treatment to avoid long-term complications, such as stricture or erosive gastritis, is imperative. Initial medical therapy entails acid suppression through proton pump inhibitor (PPI) therapy in combination with lifestyle changes [3]. PPIs are an effective first-line treatment for GERD; however, the disease is not eradicated through this therapy. Some patients require continuous maintenance therapy for recurrent symptoms. Long-term PPI use can lead to adverse effects including, but not limited to, osteoporosis, pneumonia, hypomagnesemia, acute kidney injury, dementia, and infections [4,5]. Several anatomical correction techniques have been developed to treat GERD symptoms through the reshaping of the lower esophageal sphincter (LES) [6,7,8,9,10]. Four such techniques are Nissen fundoplication (NF), transoral incisionless fundoplication (TIF), anti-reflux mucosectomy (ARMS), and Resection and Plication (RAP). NF, a procedure in which the gastric fundus is wrapped around the esophagus, is considered the gold standard treatment for GERD [11]. The TIF procedure is a minimally invasive, FDA-approved, endoscopic procedure also utilized to treat chronic GERD. The procedure entails creating a 270-degree esophagogastric wrap to anchor around the esophagus [8,9]. Over a period of a few years, the wrap created during the NF and TIF procedures can become loose, resulting in a recurrence of GERD symptoms [12,13]. ARMS and RAP are procedures that involve endoscopic mucosal resection of about 65% of the gastroesophageal junction circumference [14]. The goal of this procedure is to reduce the diameter of the gastroesophageal junction through scar formation [14]. We hypothesized that the ARMS and RAP procedures could be utilized safely and effectively to treat loose flap valves with a recurrence of GERD symptoms after prior NF and TIF procedures.

## 2. Materials and Methods

### 2.1. Selection Criteria

**Figure 1:**
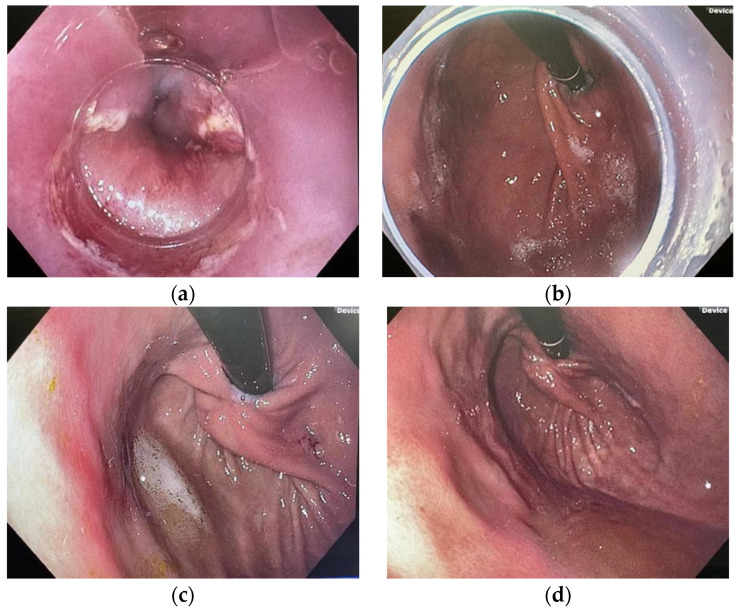
(**a**) Pre-procedural endoscopic evaluation. (**b**) Post ARMS resection. (**c**) Post ARMS resection, 6-week follow-up. (**d**) Post ARMS resection, 6-month follow-up.

We conducted a multicenter study involving four different hospitals between January 2016 and August 2020. Patients with either NF or TIF with a recurrence of GERD symptoms were included in this study (Figure 1). Specifically, eight patients had undergone the NF procedure, while ten had undergone the TIF procedure. Patients with peptic stricture, Barrett’s esophagus, high grade dysplasia, adenocarcinoma, or any contraindication to endoscopy and Endoscopic mucosal resection (EMR) were excluded. Patients with a hiatal hernia greater than 1 cm or a Hill Grade 4 valve were also excluded from this study. Informed consent was obtained from all patients.

### 2.2. Equipment

The Olympus upper endoscopes HQ180 and HQ190 were used for these procedures. Patients were placed in the left lateral position prior to the procedure. Monitored anesthesia care was used in sixteen patients and general anesthesia was used in two patients, per the recommendation of anesthesiologists.

### 2.3. Technique

1. Endoscopic evaluation of the gastroesophageal junction (GEJ) was performed.
2. A standardized procedure for mucosal resection was performed and approximately 210-to-240-degree area was resected. One centimeter of the distal esophagus and two centimeters of the cardia were resected along the lesser curve.
3. Patients were randomly selected to receive either ESD or EMR. Six consecutive patients underwent ESD while the next twelve consecutive patients underwent EMR. ESD required more time per procedure; therefore, the decision was made to switch to EMR after six cases.
4. RAP was used in nine patients with a Hill Grade 3 valve.

### 2.4. Follow Up

After the ARM/RAP procedure, our patients were given pain medications and antiemetics as needed. All patients were observed in the recovery unit for one hour. Prior to being discharged, the patients were started on a full liquid diet. Seventeen patients were discharged the same day. One patient was kept overnight because of excessive chest pain and anxiety.

A follow-up EGD was performed at 4–12-week intervals. Patients were also re-evaluated in clinic visits or by telephone after 4–12 weeks, and then again at 6, 12, 24, and 36 months after the initial procedure (Figure 1c,d).

All patients were monitored for reflux and dysphagia through follow-up questionnaires, and for stenosis through a follow-up EGD. If stenosis was found during the follow-up EGD evaluation, a gentle endoscopic dilation was performed.

A Hill Grade classification was utilized to determine the success of the procedure. A Hill Grade classification is an endoscopic grading system for esophagogastric junction competence. A normal gastroesophageal flap valve (GEFV) is classified as Hill Grade I, while an abnormal GEFV is classified as either Grade II/III/IV. A pre-procedure Hill Grade and post-procedure Hill Grade were recorded for each patient (Table 1). Success was also classified as the clinical improvement of symptoms including heartburn and regurgitation. Within-person pre–post differences in Hill Grade were examined using a *t*-test.

## 3. Results

All patients underwent the ARMS or RAP procedure successfully. Of these patients, nine patients were found to have a Hill Grade 3 Valve and nine patients had a Hill Grade 2 valve.

Two patients experienced bleeding during the procedure, and this was controlled successfully using an injection of epinephrine and a coagrasper. One patient developed bleeding two days after the procedure, and this required overnight admission and one unit of blood transfusion. Seven patients had post-procedure pain that resolved within 1–7 days. Four patients developed dysphagia after 4–6 weeks, with one requiring dilation treatment. The rest of the patients had resolution of their dysphagia after a few months. Patients were encouraged to eat a full liquid diet or soft diet, and drink water frequently during their meals. The patients on a soft diet were encouraged to eat slowly, chew their food well and drink water frequently during the meal. No other complications were noted.

A follow-up endoscopy was performed at 4–12 weeks. At follow-up, 11 patients had a Hill Grade 1 valve, seven patients had a Hill Grade 2 valve (Table 1 and Table A1) and all patients had clinical improvement in the symptoms of heartburn and regurgitation. The clinical improvement of symptoms was seen within 4–8 weeks of follow-up. The follow-up was up to 36 months. The valve grades remained the same throughout the 36 months of follow-up. A paired T-Test comparing each patient’s pre-Hill compared to post-Hill revealed the procedure produced statistically significant data (difference in means = 1.111, T = 14.5774, *p* < 0.001). All patients had improvement in symptoms and did not require PPI therapy during the follow-up evaluation.

## 4. Discussion

GERD is a common condition with a standard treatment of PPI therapy [1]. Anatomical correction of the lower esophageal sphincter with NF or TIF are established procedures for the treatment of GERD [6,7]. However, the surgically induced plication and endoscopic fundoplication can begin to loosen over time.

The ARMS procedure is a minimally invasive procedure that has shown promising clinical success [14]. The first ARMS study was published by Inoue et al. in 2014, in which they demonstrated performing a two-thirds circumferential resection of the esophagus–gastric junction [13]. The scar formation after the ARMS/RAP procedure induced narrowing of the gastric cardia opening, thus rebuilding the mucosal flap. The results of this paper revealed that there was an improvement in symptoms of heartburn and regurgitation, as measured by the Demeester score, and it revealed an overall flap grade decrease.

Our study was the first to demonstrate feasibility, efficacy and safety with minimal side effects for an ARMS/RAP procedure in patients with previous NF or TIF that has become loose over time, resulting in recurrence of GERD symptoms. After the treatment, eleven patients had a Hill Grade 1 valve and seven patients had a Hill Grade 2 valve.

The current study has notable strengths and limitations. This study introduced a novel technique that followed a reproducible protocol. In our study, ARMS/RAP was safely completed in all eighteen patients. This study did not have any patients lost to follow-up, although the population size was limited and heterogenous. This procedure was performed by one advanced endoscopist utilizing the same technique. This can be perceived as a strength or limitation, as this eliminates any technical variability, but raises questions as to whether this technique can be applied by all advanced endoscopists without modification. Furthermore, only eighteen patients were enrolled in this pilot study and clinical outcomes were only evaluated by pre- and post-Hill Grade. Thus, larger randomized control trials utilizing other means, including a GERD-HRQL questionnaire and pre- and post-procedure ambulatory pH monitoring, are needed to evaluate the procedure’s safety and efficacy.

## 5. Conclusions

In our pilot study, ARMS/RAP treatment in patients with prior NF and TIF is safe, effective, and clinically feasible, leading to significant improvement in quality of life after the procedure, in addition to patients being able to come off PPIs. Larger studies utilizing this technique with control groups and longer follow-ups are needed to further confirm the safety and feasibility of this procedure.

## Figures and Tables

**Table 1:** Pre-procedure and follow-up Hill Grades.

	Pre-Procedure	Follow-Up
Hill Grade 1	0	11
Hill Grade 2	9	7
Hill Grade 3	9	0
Total	18	18

Hill Grade I: a prominent fold of tissue along the lesser curvature next to the endoscope. Hill Grade II: the fold is less prominent and there are periods of opening and rapid closing around the endoscope. Hill Grade III: the fold is not prominent and the endoscope is not tightly gripped by the tissue.

## Data Availability

Not applicable.

## Appendix A

**Table A1:** Patient characteristics and documented pre- and post-Hill Grade valves for each patient.

Patient Number	Age	Gender	Prior Procdure	Pre-HillGrade	Post-Hill Grade
1	65	M	TIF	3	2
2	59	M	TIF	3	2
3	71	M	TIF	3	2
4	78	F	NF	3	2
5	84	M	NF	3	2
6	76	F	NF	3	2
7	73	F	TIF	3	2
8	72	F	NF	3	1
9	64	M	NF	3	1
10	68	M	TIF	2	1
11	83	M	TIF	2	1
12	63	M	TIF	2	1
13	66	F	NF	2	1
14	61	M	NF	2	1
15	67	F	TIF	2	1
16	72	F	TIF	2	1
17	58	M	NF	2	1
18	64	F	TIF	2	1

M = Male; F = female; TIF = Transoral Incisionless Fundoplication; NF= Nissen Fundoplication.
